# Protocol for the Metformin Aneurysm Trial (MAT): a placebo-controlled randomised trial testing whether metformin reduces the risk of serious complications of abdominal aortic aneurysm

**DOI:** 10.1186/s13063-021-05915-0

**Published:** 2021-12-27

**Authors:** Jonathan Golledge, Clare Arnott, Joseph Moxon, Helen Monaghan, Richard Norman, Dylan Morris, Qiang Li, Greg Jones, Justin Roake, Matt Bown, Bruce Neal

**Affiliations:** 1 Queensland Research Centre for Peripheral Vascular Disease, College of Medicine and Dentistry, James Cook University, Townsville, Queensland 4811 Australia; 2 The Department of Vascular and Endovascular Surgery, The Townsville University Hospital, Townsville, Queensland Australia; 3 The Australian Institute of Tropical Health and Medicine, James Cook University, Townsville, Queensland Australia; 4 George Institute Australia, Sydney, New South Wales Australia; 5 University of New South Wales, Sydney, New South Wales Australia; 6 Curtin School of Population Health, Faculty of Health Sciences, Curtin University, Perth, Western Australia Australia; 7 Department of Surgical Sciences, Division of Health Sciences, University of Otago, Dunedin, New Zealand; 8 Department of Surgery, University of Otago, Christchurch, New Zealand; 9 Department of Cardiovascular Services, University of Leicester, Leicester, UK

**Keywords:** Abdominal aortic aneurysm, Metformin, Randomised controlled trial, Placebo

## Abstract

**Background:**

Multiple observational studies have associated metformin prescription with reduced progression of abdominal aortic aneurysm (AAA). The Metformin Aneurysm Trial (MAT) will test whether metformin reduces the risk of AAA rupture-related mortality or requirement for AAA surgery (AAA events) in people with asymptomatic aneurysms.

**Methods:**

MAT is an international, multi-centre, prospective, parallel-group, randomised, placebo-controlled trial. Participants must have an asymptomatic AAA measuring at least 35 mm in maximum diameter, no diabetes, no contraindication to metformin and no current plans for surgical repair. The double-blind period is preceded by a 6-week, single-blind, active run-in phase in which all potential participants receive metformin. Only patients tolerating metformin by taking at least 80% of allocated medication will enter the trial and be randomised to 1500 mg of metformin XR or an identical placebo. The primary outcome is the proportion of AAA events defined as rupture-related mortality or need for surgical repair. Secondary outcomes include AAA growth, major adverse cardiovascular events and health-related quality of life. In order to test if metformin reduced the risk of AAA events by at least 25%, 616 primary outcome events will be required (power 90%, alpha 0.05).

**Discussion:**

Currently, there is no drug therapy for AAA. Past trials have found no convincing evidence of the benefit of multiple blood pressure lowering, antibiotics, a mast cell inhibitor, an anti-platelet drug and a lipid-lowering medication on AAA growth. MAT is one of a number of trials now ongoing testing metformin for AAA. MAT, unlike these other trials, is designed to test the effect of metformin on AAA events. The international collaboration needed for MAT will be challenging to achieve given the current COVID-19 pandemic. If this challenge can be overcome, MAT will represent a trial unique within the AAA field in its large size and design.

**Trial registration:**

Australian Clinical Trials ACTRN12618001707257. Registered on 16 October 2018

## Summary

Note: the numbers in curly brackets in this protocol refer to SPIRIT checklist item numbers. The order of the items has been modified to group similar items.

**Table:** 

Title {1}	Protocol for the Metformin Aneurysm Trial (MAT): A placebo-controlled randomised trial testing whether metformin reduces the risk of serious complications of abdominal aortic aneurysm.
Trial registration {2a and 2b}	ACTRN12618001707257; Registered 16^th^ October 2018.
Protocol version {3}	01 August 2021 v7.0
Funding {4}	This study receives its funding from the National Health and Medical Research Council of Australia, the Royal Australasian College of Surgeons, the Australian and New Zealand Society for Vascular Surgery, The Townsville Hospital and Health Services and the Health Research Council of New Zealand.
Author details {5a}	^1^Queensland Research Centre for Peripheral Vascular Disease, College of Medicine and Dentistry, James Cook University, Townsville, Queensland, Australia ^2^The Department of Vascular and Endovascular Surgery, The Townsville University Hospital, Townsville, Queensland, Australia ^3^The Australian Institute of Tropical Health and Medicine, James Cook University, Townsville, Queensland, Australia ^4^George Institute Australia, Sydney, New South Wales, Australia ^5^University of New South Wales, Sydney, New South Wales, Australia ^6^Curtin School of Population Health, Faculty of Health Sciences, Curtin University, Perth, Western Australia, Australia ^7^Department of Surgical Sciences, Division of Health Sciences, University of Otago, Dunedin, New Zealand ^8^Department of Surgery, University of Otago, Christchurch, New Zealand ^9^Department of Cardiovascular Services, University of Leicester, Leicester, UK
Name and contact information for the trial sponsor {5b}	James Cook University, Townsville, Australia, 4811.
Role of sponsor {5c}	The funders and sponsor had no role in the design of this protocol or the decision to submit it for publication.

## Introduction

### Background and rationale {6a}

Abdominal aortic aneurysm (AAA) rupture is an important cause of death in older adults [1]. Most AAAs are identified by incidental imaging or screening when they are small, asymptomatic and at low risk of rupture [2]. Up to 70% of small asymptomatic AAAs expand in size over time to ≥55 mm when the risk of rupture increases [2, 3]. The only established way to prevent AAA rupture is elective repair by open or endovascular surgery [4, 5]. Randomised controlled trials have shown that elective surgical repair of small (< 55 mm) asymptomatic AAAs does not reduce mortality [6]. Clinical guidelines recommend that small (< 50 mm in women and < 55 mm in men) asymptomatic AAAs are managed conservatively by repeat imaging surveillance until they reach threshold diameter when surgical repair should be considered [4, 5]. Since most non-surgically managed AAAs continue to grow in size until they reach the threshold for surgical repair, there is an unmet need to identify drug therapies able to limit AAA progression [2].

Previous randomised clinical trials have tested the efficacy of blood pressure-lowering agents [7–10], antibiotics [11–16], the mast cell inhibitor pemirolast [17], the anti-platelet inhibitor ticagrelor [18] and the fibrate fenofibrate [19], to slow AAA growth [20]. None of these trials has demonstrated a benefit of tested medications in limiting AAA growth. Important limitations of these past trials have included small sample sizes, short follow-up and a focus on imaging findings rather than clinically important outcomes [20–22].

Multiple observational studies have identified inverse associations of diabetes with AAA prevalence and AAA growth [23, 24]. These observations are contrary to the strong positive association of diabetes with other vascular diseases and their complications [25]. The Life Line Screening Study paradoxically reported that higher blood glucose levels were positively associated with the risk of AAA in people who did not have diabetes, whereas AAA risk was significantly lower for those with a confirmed diabetes diagnosis [26]. This finding suggests that the reduced prevalence of AAA in people diagnosed with diabetes may not be attributable to a protective effect of hyperglycaemia but rather may be a consequence of medication used to treat diabetes. Metformin is the longest established treatment for diabetes, and previous rodent studies have suggested that metformin limits pathological mechanisms such as inflammation and matrix remodelling implicated in AAA [27–29]. Several human observational studies have reported an association of metformin with reduced AAA growth [27, 30–32]. A meta-analysis of eight studies including 153,553 patients reported that metformin prescription was associated with a weighted mean reduction in AAA growth of 0.8 mm/year (95% confidence interval, *CI*, 0.5 to 1.1). A meta-analysis of a further three studies including 13,016 patients suggested that metformin prescription was associated with a reduction in the risk of AAA rupture-related mortality and AAA repair (AAA events; relative risk, *RR*, 0.60, 95% *CI* 0.50 to 0.71).

These data provide a strong rationale for anticipating beneficial effects of metformin in reducing the incidence of clinically important events in people with an AAA amongst whom there are no current plans for surgical repair. The Metformin Aneurysm Trial (MAT) will define the effects of metformin compared to placebo on the risk of AAA events in patients with an AAA measuring 35 mm or greater.

### Objectives {7}

The primary aim is to assess whether a daily dose of 1500 mg metformin extended release (XR) compared to placebo reduces the risk of AAA rupture-related mortality or surgical repair. The secondary aims are to assess the effects on AAA growth, major adverse cardiovascular events (MACE), health-related quality of life and requirement for peripheral vascular surgical procedures.

### Trial design {8}

**Fig. 1:**
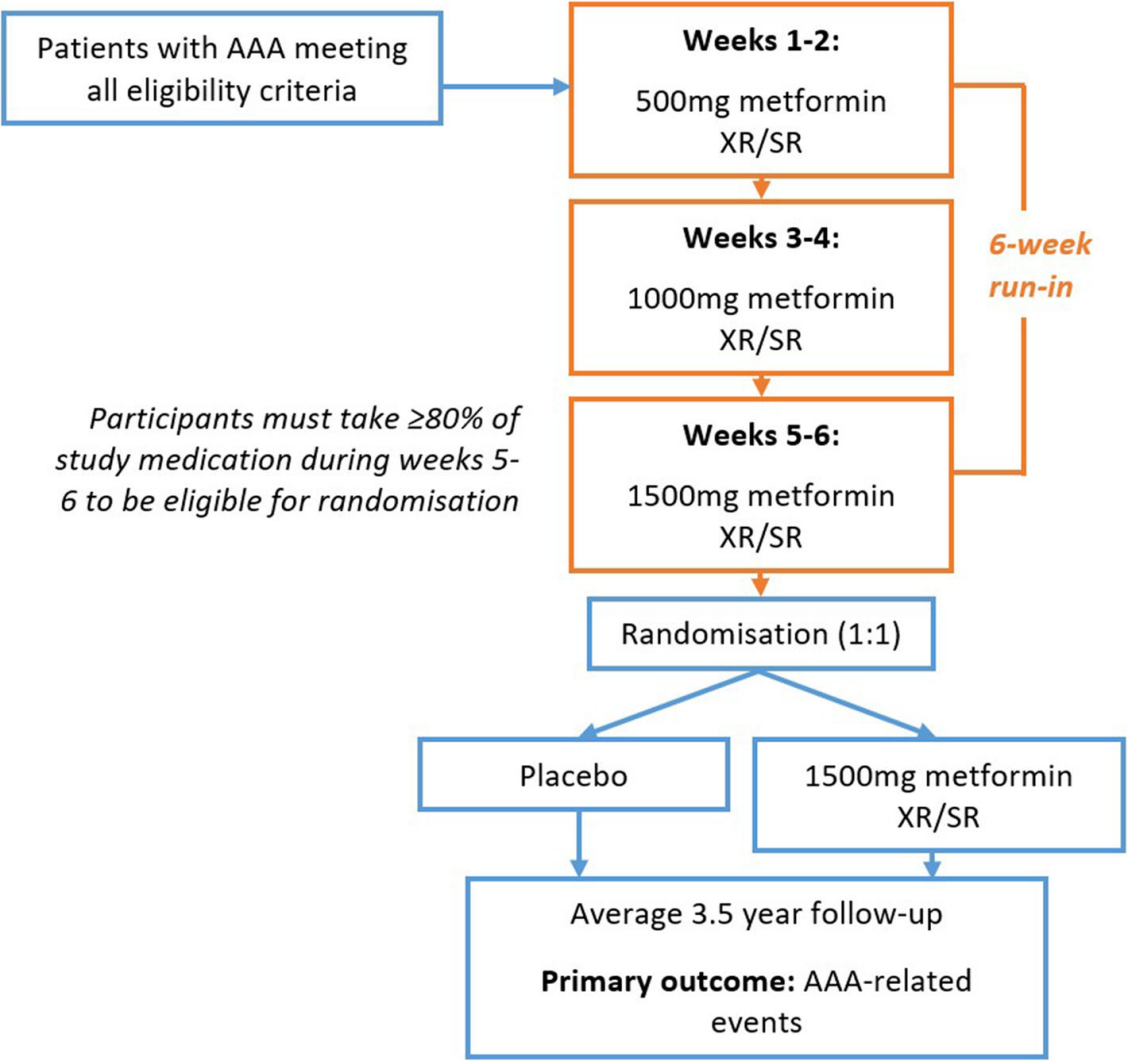
Study schema

MAT is an international, multi-centre, prospective, parallel-group, randomised, double-blind, placebo-controlled phase 3b trial. The double-blind period will be preceded by a 6-week, single-blind (participants are blinded), active run-in phase in which all potential participants receive metformin XR with fortnightly dose up-titration (Fig. 1). The aim of this run-in phase is to promote the randomisation of participants who tolerate the treatment regimen and to increase the likelihood of high treatment adherence during long-term follow-up. It is anticipated that randomised participants will receive study medication for a mean of 3.5 years. All participants will be asked to continue the study drug until the trial is completed.

## Methods: participants, interventions and outcomes

### Study setting {9}

MAT will be conducted across multiple international sites. At this stage, two sites in New Zealand and seven sites in Australia have been activated.

### Eligibility criteria {10}

#### Inclusion criteria

- An infrarenal AAA with a diameter of ≥35 mm on imaging with the treating doctor indicating that repair is not currently planned
- The participant must be at least 18 years old and provide written informed consent

#### Exclusion criteria

- Symptomatic, ruptured or infected AAA
- Previous abdominal aortic surgery including abdominal aortic bypass, endarterectomy or repair
- Contraindications to metformin, including:
  - Renal impairment (eGFR < 45 ml/min/1.73m^2^)
  - Liver cirrhosis (current or previous diagnosis)
  - Severe heart failure: defined as New York Heart Association Class IV, requiring in-patient treatment within the last 12 months or leading to shortness of breath at rest
  - Binge alcohol use defined as consuming 5 or more drinks (male), or 4 or more drinks (female), in 2 h
  - Previous allergic reaction to metformin [33–35]
- Current indication for metformin (i.e. diabetes defined by HbA1c ≥6.5%) [36]
- Involvement in another drug trial
- Terminal illness

### Who will take informed consent {26a}

Written informed consent must be obtained in accordance with Good Clinical Practice (GCP) from all participants before conducting any study procedures including screening assessments. Consent will be obtained by an approved researcher with appropriate training in good clinical practice. This will be obtained prior to screening. The participant information sheet and consent form (PISCF) signed by the participant and the authorised person conducting the consent process must be the current ethics committee (EC)/institutional review board (IRB)-approved version. The complete original signed (PISCF) must be filed in the patient’s study file. A copy must be provided to the participant. Prospective participants will be informed that the screening and run-in phase will determine eligibility for the study and that signing the PISCF does not guarantee enrolment into the study.

### Interventions

#### Explanation for the choice of comparator {6b}

The comparator will be inactive placebo identical to the intervention in all aspects. This comparator has been chosen since currently no medication has been shown to be effective in limiting AAA progression.

#### Intervention description {11a}

Participants who successfully complete the run-in phase and who meet eligibility criteria for study continuation will be randomised to receive:

i. Metformin XR: 3 × 500 mg tablets per day
  *Or*
ii. Matched placebo: 3 × 500 mg tablets per day

The study drug will be administered orally once daily with water. Participants will be advised to take the study drug with their evening meal. If a dose is missed, participants will be advised to take the study drug as soon as they remember. However, it will be made clear to participants that a double dose should not be taken on the same day. The study treatment is double-blinded.

#### Criteria for discontinuing or modifying allocated interventions {11b}

Participants that develop intolerance to study treatment during the trial will be able to down-titrate to 1 or 2 tablets (500–1000 mg) per day as required without unblinding. Participants, their physicians, investigators and adjudication committee will be blinded to treatment allocation for the duration of the trial. Unblinding will be possible where clinically required using a dedicated Web-based function. Only the Data and Safety Monitoring Board (DSMB) will have routine access to unblinded data prior to trial completion. The study drug will be provided to participants using a pharmacy dispensing service.

#### Strategies to improve adherence to interventions {11c}

The following procedures will be implemented to maximise participants’ adherence to the study treatment:

- Participant education during all follow-up interactions which will focus on the importance of taking the study drug, including timing, storage and what to do in the event of a missed dose
- Participants will be asked at each follow-up how regularly they take the study drug (every day [about 100% of tablets], nearly every day [about 80–99% of tablets], some days [about 40–79% of tablets], a few days [about 10–39% of tablets], almost never [about 1–9% of tablets] or never [0%]). Reasons for poor or non-adherence will be sought and addressed as far as possible. Participants will also be asked at each follow-up how many tablets are remaining in the bottle/kit
- Participants will be provided with the contact details of the responsible researcher so that they can make contact if for any reason they are unable to continue their study drug or have missed multiple doses and are unsure whether to continue

### Relevant concomitant care permitted or prohibited during the trial {11d}

Patients will receive medical management to reduce their cardiovascular event risk and management of any co-morbidities by their treating physician according to current practice guidelines [4, 5].

### Provisions for post-trial care {30}

Following completion of the study, participants will be referred to their primary care provider and treating physician for ongoing management. Post-study follow-up of all participants (off treatment) using electronic registry data will continue for at least 5 years after the database lock, subject to funding availability to study longer term outcomes. No arrangements are in place to provide metformin to participants after the study has completed.

### Outcomes {12}

#### Primary outcome

The primary assessment will compare the number of AAA events (defined as the occurrence of AAA repair or AAA mortality due to rupture) between study groups which were observed during the treatment period. This will be in the form of an intention to treat (ITT) comparison including all randomised participants.

#### Secondary outcomes

Secondary assessments will involve ITT comparisons including all randomised participants to test the effect of metformin versus placebo on the following outcomes:

i *AAA growth assessed by ultrasound*: Current clinical guidelines in every participating country recommend patients with ≥35 mm AAA undergo ultrasound imaging surveillance as part of their standard clinical care [5, 37, 38], which will be used to determine AAA growth over the course of the study. Digital Imaging and Communications in Medicine (DICOM®) AAA ultrasound images will be transferred from participating sites to the coordinating centre at screening and thereafter. The maximum anteroposterior orthogonal outer wall-to-outer wall measurement will be centrally assessed by experienced observers who have previously been shown to have excellent imaging analysis reproducibility. If DICOM images are not available, the maximum anteroposterior orthogonal outer wall-to-outer wall measurement will be measured by a site investigator experienced in reading ultrasound. Linear mixed effects modelling will be used to analyse AAA growth as previously described [39, 40]. If participants undergo imaging by other or additional modalities as part of clinical care, such as CT and/or magnetic resonance imaging, these images will also be collected and used to assess AAA diameter and volume growth independent of ultrasound imaging. If data becomes available from these differing imaging modalities on a sufficient sample of participants, a meta-analysis of the different data sets will be performed.
ii *MACE*: Defined as non-fatal myocardial infarction, non-fatal stroke and cardiovascular death (i.e. sudden death, death due to myocardial infarction, valvular heart disease, cardiomyopathy or primary arrhythmia, or other cardiovascular disease or investigations or procedures related to these presentations).
iii *Health-related quality of life*: Health-related quality of life will be assessed by generic (short form 36; SF-36) and disease-specific (the Aneurysm-Dependent Quality of Life [AneurysmDQoL]). These assessments will be performed at entry and annually.
iv *Requirement for the peripheral vascular surgical procedure*: Defined as lower limb peripheral revascularisation (open or endovascular), carotid artery revascularisation, other aneurysm repair and major amputation. Incidence of the composite event (i.e. first occurrence of any procedure) and total number of events will be examined
v *All-cause mortality* is defined as death from any cause.

#### Exploratory outcomes

Exploratory assessments will investigate other possible beneficial or adverse effects of metformin during the treatment period as well as during planned post-trial follow-up. Exploratory assessments will involve ITT analyses amongst all randomised participants of the effects of allocation to metformin versus placebo during the study period on outcomes including, but not limited to:

i Cause-specific mortality including deaths from cardiovascular disease and deaths from cancer and different types of cancers
ii Development of diabetes mellitus
iii Cancer at all sites (excluding any known to pre-date randomisation and non-melanoma skin cancers) specifically including lung cancer, bowel cancer, bladder cancer and prostate cancer
iv Symptomatic gout. A tertiary analysis will be performed to examine whether participants randomised to metformin have fewer episodes of gout. Prior observational evidence suggests that metformin could prevent exacerbations of gout through anti-inflammatory effects [41]
v Circulating biomarkers of AAA pathology will be assessed in a subgroup of participants who consent and where it is feasible to obtain blood samples for a biomarker sub-study
vi Requirement for repeat open or endovascular AAA repair (i.e. re-intervention)

#### Safety assessments

Safety assessments will be performed on the per-protocol and ITT datasets of the effects of allocation to metformin versus placebo during the study period on:

i Total serious adverse events
ii Lactic acidosis
iii Symptomatic hypoglycaemia

#### Health economic assessments

If a significant effect of metformin on the primary outcome is observed, cost-effectiveness and cost-utility analyses will be performed. These evaluations will adopt a health system perspective, including estimates of all healthcare costs, particularly those relating to drugs, doctors’ visits and in-patient charges and costed using standard country-specific list prices. The cost-effectiveness analysis will produce an incremental cost-effectiveness ratio defined as a cost per AAA event avoided. For the cost-utility analysis, health utility scores will be generated for each participant by converting responses to the SF-36 into a single SF-6D score using validated algorithms and applying country-specific utility weights where available; weights currently exist for the UK and Australia. Each year participants will be asked how many outpatient appointments they have attended, and specifically how many of those appointments were related to their AAA. These analyses will generate costs per quality-adjusted life year (QALY) gained (or lost) and include sensitivity analyses, particularly around using pooled or country-specific data for each cost-utility analysis. Country-specific thresholds for determining cost-effectiveness will be used where available and presented in combination with cost-effectiveness acceptability curves.

### Adjudication

An independent adjudication committee, blinded to study treatment assignment, will adjudicate all potential primary outcome events as well as myocardial infarction, stroke and all-cause mortality.

### Participant timeline {13}

A summary of all study assessments is presented in Table 1.

**Table 1:** Schedule of evaluations

Visit	Screening −10 to −6 weeks	Run-in^**a**^ −6 to 0 weeks	Randomisation 0 weeks	Follow-up^**b**^			
				3 monthly	6 monthly	Annually	Final^**c**^
Time window for evaluation	N/A	+ 2 days	N/A	±14 days	±14 days	±14 days	N/A^c^
Informed consent (prior to or at screening)	X						
Assessment of eligibility	X						
Demographics	X						
Anthropometrics—height and weight	X					X^f^	X^f^
Behavioural—smoking	X						
Medical history	X						
Concomitant medications	X					X	X
AAA imaging (standard care)^h^	X					X	X
Blood tests—creatinine, eGFR, HbA1c, CRP (if available), LDL-C (if available)^d^	X					X^g^	X^g^
Blood sample for biomarker sub-study^e^	X					X^e^	X
QoL questionnaires: SF-36, AneurysmDQoL	X					X	X
Run-in medication dispensed	X						
Randomisation			X				
Study medication dispensed			X		X	X	
Study medication adherence		X		X	X	X	X
AESI reporting		X		X	X	X	X
Serious adverse event reporting		X		X	X	X	X
Assessment of exploratory outcomes not classified as SAEs (diagnosis of cancer, diagnosis of diabetes, symptomatic gout)				X	X	X	X
Health economic assessment questions						X	X

^a^Participants will be contacted by telephone fortnightly during the 6-week run-in period

^b^Participants will be contacted every 3 months after randomisation

^c^Participants will undergo a final follow-up within 3 months of the date of the last adjudicated primary outcome event

^d^If blood tests are available from within 6 months prior to screening, no need to repeat tests at screening

^e^Only for participants who have consented to the biomarker sub-study. Samples will be collected at entry, at years 1 and 3, and at the final scheduled follow-up

^f^Weight only

^g^eGFR only. If blood tests are available from within 3 months prior to annual follow-up/final follow-up, no need to repeat tests

^h^If imaging is available within 12 months of screening/annual follow-up, no need to repeat tests

### Sample size {14}

While the trial is outcome event driven (an estimated 616 primary outcome events are needed), a sample size estimate has considered the likely participant numbers needed. The planned sample size is 1954 (metformin *n* = 977; placebo *n* = 977) which will provide 90% power (*p* = 0.05) to detect a 25% or greater reduction in the relative risk of the primary outcome with metformin compared to placebo over a mean follow-up of 3.5 years. This calculation was based on:

- A 25% relative risk reduction for the primary outcome which is less than reported by observational studies comparing AAA events between patients who were, or were not receiving metformin [42]
- An AAA repair or rupture rate of 9% per annum (31% over 3.5 years) in the control group has been assumed to accrue a total of 616 primary endpoint events in the trial. This event rate is lower than the occurrence of AAA repair or rupture in our prospective registry (AAA events observed at 12% per annum) [42] and less than that reported in a number of small AAA trials [2, 43]. For example, the UK small aneurysm trial and the surveillance versus aortic endografting for small aneurysm (CAESAR) trial reported 3-year rates of AAA rupture or repair of 52% and 61%, respectively [43]
- A drop-out rate of 5% per annum (discontinuation of metformin in the intervention group) which is expected to be minimised by the active run-in period
- A drop-in rate of 1% per annum (commencement of metformin in the control group) which is expected to be low because incident diabetes will be uncommon and options for use of therapy based on agents other than metformin will be discussed with participants and their responsible physician

### Recruitment {15}

Potential participants will be identified and sought from three main sources: existing AAA registries, collaborating hospitals and vascular departments and community sources and collaborations. We will also work with every site to explore other databases held by imaging services and research groups.

#### Registry recruitment

Previous trials demonstrate that registry-based recruitment methods can facilitate the recruitment of many thousands of participants over short periods of time in a cost-effective manner [44]. Study invitation packages, including a letter of invitation (LOI), study brochure and a postage-paid return envelope, will be mailed out to potentially eligible individuals. About 7–10 days after mailing the study invitation or on receipt of a returned and completed LOI (except when a LOI indicates an individual does not want to be contacted), a staff member will call the individual to confirm interest in study participation, assess potential eligibility and answer any questions they may have about the trial. Suitable and interested participants will be consented and enter screening.

#### Collaborating hospitals and vascular departments

Sites that do not have existing AAA registries may identify lists of patients who have an AAA, no prior repair and no diabetes using locally approved methods. This may include identifying patients from vascular laboratory records and electronic medical records. Potential participants may also be identified during routine follow-up with their specialist. Site staff will invite potential participants to the study either in person, by phone or by mail using a combination of the study brochure and LOI. Potential participants who are interested will be consented by appropriately delegated staff and enter screening. For sites that do not have study personnel available, and with appropriate regulatory approvals, and if required participant consent, the collaborating hospitals will provide the coordinating centre with the contact details of potential participants via a secure Web-based system. A study staff member at the coordinating centre will then invite the participants to take part in the study.

#### Community sources and collaborations

To complement these recruitment methods, private medical and surgical centres, general practitioners and practice nurses will also be able to offer a study invitation pack to potential participants when they are seen for routine care in their clinic, or by mail. In addition, investigators will approach large health organisations to promote the study and provide information about how health professionals can help their eligible patients join the study. Lastly, recruited participants will be able to recommend friends or relatives who they think may be eligible and interested in taking part in the study and other potential participants may volunteer themselves if they hear about the study from other sources, such as approved advertisements or the coordinating centre website regarding the trial.

### Assignment of interventions: allocation

#### Sequence generation {16a}

A random sequence for study arm allocation will be generated by the trial statistician prior to commencement. Randomisation will be conducted using a secure Web-based system and will be stratified by study centre, sex and AAA diameter (35–38.9, 39–42.9, 43–46.9 and ≥ 47 mm) on imaging. Randomisation will be blocked in a 1:1 ratio.

#### Concealment mechanism {16b}

The trial statistician will also generate a sequence of unique codes for every active/placebo drug kit. The drug kit codes will be provided to the approved Investigational Medicinal Product (IMP) manufacturer who will ensure that study drug and placebo packs are labelled appropriately and that the study team, pharmacy staff, investigators and participants are blinded to treatment allocation.

#### Implementation {16c}

Each drug kit will contain a 6-month supply of study medication and will be identified by a kit number only. Study medication will be dispensed by the study pharmacist at baseline and 6 monthly thereafter. The kit numbers allocated to participants will be revealed to the study pharmacist on entry of the participant’s ID into the online database management system. For each participant, a new kit code will be generated every 6 months that will correspond with the treatment group the participant has been allocated.

### Assignment of interventions: blinding

#### Who will be blinded? {17a}

All study team members and participants will be blinded to the allocation. The IMP manufacturer will be unblinded to allocation and will generate active drug and placebo which will be identical in appearance and will be concealed by identical packaging, labelling and administration scheduling.

#### Procedure for unblinding if needed {17b}

Emergency unblinding can take place at any time using the secure Web-based system. It will occur for any participant experiencing a serious adverse event (SAE) for which the clinical management of the SAE will be facilitated by the unblinding of the participant’s treatment allocation. The principal investigator will make this decision. It is anticipated that for the majority of instances, appropriate clinical management can proceed with the assumption that the participant has been treated with metformin without needing to unblind the participant. The main reason for unblinding will be in the case of a suspected unexpected serious adverse reaction (SUSAR).

### Data collection and management

#### Plans for assessment and collection of outcomes {18a}

All data will be collected by members of the study team as described in the delegation log. During screening and follow-up, information that is obtained directly from participants may be confirmed from retrospective sources including medical records. In the case of safety monitoring and reporting, confirmation will again be sought about participants’ health status from medical records. All clinical and laboratory-related information will be recorded in a de-identified format using the participant’s unique trial identifier.

The quality of life questionnaires (SF-36 and AneurysmDQoL) have been validated for use in AAA patients. The SF-36 has the advantage of being able to be converted to the SF-6D for a cost-utility analysis [45]. The AneurysmDQoL is an individualised measure of the impact of AAA on patients’ quality of life, containing 23 domains with two overview items to assess the overall quality of life and impact of AAA on quality of life [46].

Source documents for the study constitute consent forms, electronic case report forms (eCRF), information obtained on reported outcome measures, death certificates, pathology results, DICOM ultrasound images and imaging reports and drug supply records. These will be retained for at least 15 years from the completion of the study.

#### Plans to promote participant retention and complete follow-up {18b}

Participants that discontinue study medication will be encouraged to remain on the study protocol, and with their consent, continue to be followed and their data included in the final analysis in accordance with an ITT analysis.

#### Data management {19}

All study data collected for outcome analysis will be completed via a secure password-protected Web-based data management system hosted by IBM Corp, USA. This will allow for real-time data query generation for values entered outside of pre-set valid ranges and consistency checking. This system will facilitate data reporting and assist overall trial management for all participating centres. Data entry will be performed at participating sites, with the exception of sites that do not have study personnel available. In this case, data will be entered by the coordinating centre. Only authorised staff will have access. All entered data forms will be electronically signed by authorised study staff. All changes made following the initial entry will have an electronically dated audit trail. Centralised coding of outcomes will be performed by a trained medical coder and reviewed by the monitor, to confirm the accuracy of coding and correct reporting of outcomes by sites.

#### Confidentiality {27}

Every precaution will be taken to respect the privacy of participants in the conduct of the study. Only de-identified data will be entered into the secure Web-based data management system to maintain participant confidentiality. All individual and site information will be de-identified in reporting data and results to protect the confidentiality of participants. Only approved investigators will have access to data.

#### Plans for collection, laboratory evaluation and storage of biological specimens for genetic or molecular analysis in this trial/future use {33}

A sub-set of participants may consent to the MAT biomarker sub-study which involves exploratory biomarker analysis. Individuals who consent to take part in the additional sub-study will be required to have 20 ml of blood taken including EDTA plasma, citrate plasma, PAX Gene tube, serum and whole blood. Samples will then undergo cooling, centrifugation, separation into cryovials and storage at below −80 °C. These samples will be used to assess relevant biomarkers including D-dimer, matrix metalloproteinases, osteoprotegerin and osteopontin. Samples will be collected at entry, at years 1 and 3 and at the final scheduled follow-up.

#### Additional consent provisions for collection and use of participant data and biological specimens {26b}

A sub-set of participants from sites with resource for blood sampling may consent to the MAT biomarker sub-study which involves exploratory biomarker analysis. Individuals who consent to take part in the additional sub-study will be required to donate 20 ml of blood (including EDTA plasma, citrate plasma, samples for RNA analysis (PAX Gene tube, Qiagen), serum and whole blood). Samples will then undergo cooling, centrifugation, separation into cryovials and storage at below −80 °C. These samples will be used to assess biomarkers relevant to AAA progression including D-dimer, matrix metalloproteinases, osteoprotegerin and osteopontin. Samples will be collected at entry, at years 1 and 3 and at the final scheduled follow-up.

### Statistical methods

#### Statistical methods for primary and secondary outcomes {20a}

All participants who have been randomised will be included in the primary analysis and will be analysed according to their randomly allocated treatment [47]. Statistical analyses will be conducted according to a detailed pre-specified data analysis plan which will be developed prior to completion of the trial and unblinding of data. A brief account of the statistical methods is included here. For the primary outcome, a time-to-event analysis will be conducted to test our hypothesis that metformin will reduce the primary endpoint. The hazard ratio (HR) and 95% CI will be calculated using the Cox proportional hazard analysis, and event risk will be plotted on a Kaplan-Meier graph. A *p*-value < 0.05 will be considered significant. The focus of the trial will be on the primary hypothesis, but we will also examine the effect of metformin on secondary and tertiary outcomes. A detailed statistical analysis plan will be prepared before completion of the trial.

#### Interim analyses {21b}

No formal interim analyses are planned; however, the Data and Safety Monitoring Committee (DSMC) will be charged with advising the Management Committee of the need to update the trial design or terminate the trial early based on clear evidence of harm or efficacy prior to scheduled completion.

#### **Methods for additional analyses (e.g. subgroup analyses) {20b}**

Subgroup analyses will consider the effect of age, sex, recruitment country, smoking, coronary heart disease, peripheral artery disease, initial AAA diameter and HbA1c on the primary outcome.

#### **Methods in analysis to handle protocol non-adherence and any statistical methods to handle missing data {20c}**

A per-protocol analysis is planned focused on participants who regularly took the study drug every day or nearly every day.

#### **Plans to give access to the full protocol, participant-level data and statistical code {31c}**

It is envisaged that the datasets analysed during the current study will be available from the corresponding author on reasonable request although approval for this has not been obtained at this point.

### Oversight and monitoring

#### Composition of the coordinating centre and trial steering committee {5d}

MAT is an investigator-initiated and investigator-conducted study. MAT is managed by a Central Coordinating Centre based at the Queensland Research Centre for Peripheral Vascular Disease (QRC-PVD) at James Cook University in Townsville, Australia, and supported by The George Institute for Global Health in Sydney, Australia. The study is overseen by a Steering Committee (SC) comprised of experienced investigators, healthcare providers, statisticians, trialists and epidemiologists with appropriate clinical and research expertise relevant to the design and conduct of MAT. The steering committee is responsible for the execution of the study design, protocol, data collection and analysis plan, as well as publications. The SC has the right to appoint new members and co-opt others to add to the integrity of the conduct of the study and analyses. The SC is co-chaired by Professor Jonathan Golledge and Professor Bruce Neal.

#### Composition of the data monitoring committee, its role and reporting structure {21a}

The DSMC will monitor safety outcomes for potential harmful effects and provide reports to the SC on recommendations to continue, modify or halt the study. The DSMC will be governed by a charter that will outline their responsibilities, procedures and confidentiality. They will review unblinded data from the study at regular intervals during follow-up. The first meeting will be held within 6 months after the start of recruitment.

### Adverse event reporting and harms {22}

#### Adverse events (AE)

An AE is defined as any untoward medical occurrence in a patient or clinical investigation subject administered a pharmaceutical product at any dose and which does not necessarily have a causal relationship with this treatment. Therefore, an AE can be any unfavourable and unintended sign (including an abnormal laboratory finding, for example), symptom or disease temporarily associated with the use of an investigational product. This definition includes intercurrent illness or injuries and an exacerbation of a pre-existing condition.

#### Adverse events of special interest (AESI)

Since the safety profile of metformin is well established, only adverse events of special interest (AESI) will be collected during the course of the MAT study. This will include symptomatic hypoglycaemia and lactic acidosis.

#### Serious adverse events (SAEs)

A SAE is defined as any untoward medical occurrence that at any dose:

- Results in death
- Is life-threatening in the opinion of the attending clinician (i.e. the patient was at risk of death at the time of the event; it does not refer to an event that might hypothetically have caused death if it had been more severe)
- Requires inpatient hospital admission or prolongation of existing hospital stay. Any hospital admission that was planned prior to randomisation will not be reported as an SAE
- Results in persistent or significant disability or incapacity
- Results in congenital anomaly or birth defect
- Is an important medical event in the opinion of the attending clinician (i.e. not life-threatening or resulting in hospital admission, but may jeopardise the participant or require intervention to prevent one or other of the outcomes listed above)

All SAEs are required to be reported via the Web-based data management system within 24 h of the study team first becoming aware of the event by reporting the event in the eCRF. SAEs are also required to be reported to the site investigator and to the relevant EC/IRB and/or sponsor in accordance with and within the timeframes specified in the relevant committee guidelines.

AEs that do not fall into these categories are defined as non-serious. If treatment is discontinued as a result of any AE, serious or non-serious, the study team will document all events leading to the discontinuation of treatment. SAEs will be collected from the time a participant gives written consent until completion of the study. If an SAE is unresolved at the conclusion of the study or if the participant withdraws early from the study, a clinical assessment will be made by the investigator as to whether continued follow-up of the SAE is warranted.

#### Suspected unexpected serious adverse reactions (SUSAR)

An unexpected adverse reaction (UAR) is an adverse reaction, the nature or severity of which is not consistent with the applicable product information. A SUSAR is any UAR that at any dose meets the definition of an SAE. All SUSARs will be reported to regulatory authorities in accordance with country-specific requirements and in compliance with *International Council on Harmonisation* (*ICH*) *Clinical Safety Data Management: Definitions and Standards for Expedited Reporting*.

### Frequency and plans for auditing trial conduct {23}

Prior to commencement of the study at any participating centre, all designated research staff including the PI, co-investigator(s) and research nurse(s)/coordinator(s) will be trained in the study procedures. Data monitoring will take a risk-based approach and involve a combination of remote, online and on-site monitoring. At least once per year, representatives of the coordinating centre will conduct a monitoring visit to all participating centres by video-conference, or if feasible or required on-site visits will take place. Study monitoring will ensure that the study is being conducted in accordance with the protocol, ICH GCP Guidelines, and meets relevant ethical and regulatory requirements. The monitor will verify patient consent and eligibility and review relevant source documents according to a detailed monitoring plan available as a separate document. At completion of the study, the monitor will ensure that there are plans in place for the long-term storage of all the relevant data and source documentation (for a minimum of 15 years or a longer period if required by applicable regulatory requirements). The study may be audited by third parties and inspected by government regulatory authorities. CRFs, source documents and other study files must be accessible at all study sites at the time of auditing and inspection during the course of the study and after completion of the study.

### Plans for communicating important protocol amendments to relevant parties (e.g. trial participants, ethical committees) {25}

This study will be carried out according to ICH GCP Guidelines, the NHMRC National Statement on Ethical Conduct in Research Involving Humans (1999) and the Notes for Guidance on Good Clinical Practice as adopted by the Therapeutic Goods Administration (2000) (CPMP/ICH/135/95), applicable local regulations including the EU Clinical Trial regulation and with the ethical principles laid down in the World Medical Associations Declaration of Helsinki. The protocol, PISCF, any material to be given to prospective participants and any subsequent modifications will be reviewed and approved by the EC or IRB responsible for oversight of the study. Prior to study commencement, investigators are required to sign a protocol signature page confirming his/her agreement to conduct the study in accordance with these documents and all the instructions and procedures contained in this protocol and to give access to all relevant data and records to monitors, auditors, IRB/ECs and regulatory authorities as required. If an inspection of the clinical site is requested by a regulatory authority, the investigator must inform the coordinating centre immediately.

An amendment is defined as a written description of change(s) to or formal clarification of a study protocol which may impact the conduct of the clinical study, potential benefit of the clinical study, or may affect subject safety, including changes of study objectives, study design, subject population, sample size, study procedures or significant administrative aspects. An administrative change is defined as a minor correction or clarification that has no significant impact on the way the clinical study is conducted and no effect on subject safety. Protocol amendments must be approved by the Steering Committee, Regulatory Authorities where required and the EC/IRB. In cases where the amendment is required in order to protect the subject safety, the amendment can be implemented prior to the EC/IRB approval. Notwithstanding the need for formal approval of a protocol amendment, the investigator is expected to take any immediate action required for the safety of any subject included in the study, even if this action represents a deviation from the protocol. In such cases, the coordinating centre should be notified of this action and the relevant EC/IRB informed within 72 h.

### Dissemination plans {31a}

The study protocol and results of this study will be published in peer-reviewed journals and made available to all participants, investigators and the research community. The study protocol and/or results may be used in conference presentations nationally and/or internationally. Individual results of participants will not be published or disseminated. Authorship will be granted to individuals making a substantial contribution to the design, initiation or conduct of the trial and/or analysis and interpretation of trial data.

## Discussion

An estimated 20 million people worldwide have an AAA [48]. AAA rupture is believed to be responsible for approximately 200,000 deaths each year globally [1]. AAAs are typically identified when they are small and an effective treatment given at this stage would prevent or limit the risk of AAA rupture and the need for surgery [21, 22]. Surveys suggest that identification of effective medications to limit the need for surgery and AAA rupture is a top priority for both patients and vascular specialists [49, 50]. Despite an enormous number of pre-clinical studies and multiple clinical trials, there is no convincing evidence to recommend any drug therapy for AAA [20, 21]. Based on strong pre-clinical and human observational data, this trial will test whether metformin limits serious AAA-related events. Unlike all prior trials, MAT is powered to test the effect of metformin on AAA events.

Metformin has an established safety profile in people with diabetes [51]. Multiple previous randomised trials suggest that metformin administration is also safe in people without diabetes. The GIPS-III placebo-controlled trial for example reported similar rates of SAEs in metformin (3%) and placebo (2%) groups amongst a population who had an acute myocardial infarction but no diabetes [52, 53]. Renal function and glucose concentrations were similar in both groups [52, 53]. Another trial of 203 young women without diabetes randomised to 1500 mg of metformin or placebo daily found no excess of SAEs in the intervention group [54]. Metformin administration has now been investigated in a range of populations that do not have diabetes with excellent safety reported [52–61]. Thus, there is a strong expectation that metformin is safe to administer in people without diabetes as planned in MAT.

Several other trials investigating the efficacy of metformin in limiting AAA progression have commenced or are planned [62, 63]. A key difference in the design of MAT compared to these other trials is that the primary outcome is the rate on AAA events not AAA growth. Potential advantages of the chosen endpoint over AAA growth measurement include greater clinical relevance, easier determination and stronger subsequent implementation impact. In addition, AAA growth is difficult to model accurately and exhibits temporal and inter-patient variation [22, 64, 65]. While decisions about the requirement for AAA repair may vary between vascular surgeons, investigational sites and countries, the blinded allocation of treatment and the stratification of randomisation by site will be protected against bias from these potential issues. Furthermore, all suspected primary outcome events will be adjudicated by review of hospital notes and other relevant materials by a blinded endpoint adjudication committee comprised of experts in the management of AAA.

A number of challenges and potential limitations of the design of MAT are also noted. Firstly, the study design requires a much larger sample size than all prior AAA drug trials [20]. This requires international collaborative design and funding support from multiple bodies which is challenging to achieve particularly during the current COVID-19 pandemic. The event rate chosen for the sample size estimate is believed to be conservative but could vary from prior trials and across nations which would influence the sample needed. While the trial has been designed to include participants tolerant of metformin this may wane over time and one previous trial, which did not include a run-in phase, reported premature discontinuation of metformin in 30% of participants [66].

In conclusion, MAT is designed to be the largest clinical trial to test a medical treatment for AAA. A positive finding from MAT will identify metformin as the first AAA drug effecting at preventing AAA-related death or rupture. Since metformin is low cost, safe and available worldwide, the trial will have direct clinical implications for people with small AAAs around the world for whom no preventive therapy is currently available.

## Trial status

Commenced recruitment 1st November 2020.

Aim for completion of recruitment December 2025.

Protocol version 7.0 1st August 2021.

## Abbreviations

AMPK: 5′ Adenosine monophosphate-activated protein kinase
AAA: Abdominal aortic aneurysm
AE: Adverse events
AESI: Adverse events of special interest
AneurysmDQoL: Aneurysm-Dependent Quality of Life
DSMB: Data and Safety Monitoring Board
DMC: Data monitoring committee
DICOM®: Digital Imaging and Communications in Medicine
eCRF: Electronic case report forms
EC: Ethics committee
XR: Extended release
GCP: Good Clinical Practice
HR: Hazard ratio
IRB: Institutional review board
ITT: Intention to treat
ICH: International Council on Harmonisation
IMP: Investigational Medicinal Product
LOI: Letter of invitation
MACE: Major adverse cardiovascular events
MAT: Metformin Aneurysm Trial
PICSF: Participant information sheet and consent form
QALY: Quality-adjusted life year
QRC-PVD: Queensland Research Centre for Peripheral Vascular Disease
SAE: Serious adverse event
SC: Steering Committee
SUSAR: Suspected unexpected serious adverse reactions
UAR: Unexpected adverse reaction
